# Importance of medical data preprocessing in predictive modeling and risk factor discovery for the frailty syndrome

**DOI:** 10.1186/s12911-019-0747-6

**Published:** 2019-02-18

**Authors:** Andreas Philipp Hassler, Ernestina Menasalvas, Francisco José García-García, Leocadio Rodríguez-Mañas, Andreas Holzinger

**Affiliations:** 1 Holzinger Group, HCI-KDD, Institute for Medical Informatics/Statistics, Medical University Graz, Graz, 8036 Austria; 2 Institute of Interactive Systems and Data Science, Graz University of Technology, Graz, 8010 Austria; 3 Center for Biomedical Technology, Universidad Politecnica de Madrid, Madrid, 28000 Spain; 4 Division of Geriatric Medicine, Virgen del Valle Geriatric Hospital, Toledo, 45000 Spain; 5 Division of Geriatric Medicine, University Hospital of Getafe, Getafe, 28905 Spain

**Keywords:** Health data analytics, Data mining, Machine learning, Predictive modeling, Risk factor discovery, Data preprocessing, Missing value imputation, Frailty syndrome

## Abstract

**Background:**

Increasing life expectancy results in more elderly people struggling with age related diseases and functional conditions. This poses huge challenges towards establishing new approaches for maintaining health at a higher age. An important aspect for age related deterioration of the general patient condition is frailty. The frailty syndrome is associated with a high risk for falls, hospitalization, disability, and finally increased mortality. Using predictive data mining enables the discovery of potential risk factors and can be used as clinical decision support system, which provides the medical doctor with information on the probable clinical patient outcome. This enables the professional to react promptly and to avert likely adverse events in advance.

**Methods:**

Medical data of 474 study participants containing 284 health related parameters, including questionnaire answers, blood parameters and vital parameters from the Toledo Study for Healthy Aging (TSHA) was used. Binary classification models were built in order to distinguish between frail and non-frail study subjects.

**Results:**

Using the available TSHA data and the discovered potential predictors, it was possible to design, develop and evaluate a variety of different predictive models for the frailty syndrome. The best performing model was the support vector machine (SVM, 78.31%). Moreover, a methodology was developed, making it possible to explore and to use incomplete medical data and further identify potential predictors and enable interpretability.

**Conclusions:**

This work demonstrates that it is feasible to use incomplete, imbalanced medical data for the development of a predictive model for the frailty syndrome. Moreover, potential predictive factors have been discovered, which were clinically approved by the clinicians. Future work will improve prediction accuracy, especially with regard to separating the group of frail patients into frail and pre-frail ones and analyze the differences among them.

**Electronic supplementary material:**

The online version of this article (10.1186/s12911-019-0747-6) contains supplementary material, which is available to authorized users.

## Background

Demographic predictions for the 21^*st*^ century [1] show a new scenario characterized by a modest increase in life expectancy, but a significantly greater burden of disability, which will increase the demand for health and care costs and challenge the sustainability of the system. Both the aging of the population and the growth of the population are driving the increase in Disability Adjusted Life Years (i.e. DALYs) due to the burden of non-communicable diseases in older ages, associated with an increase in years lived with disability. According to the last Global Burden of Disease (2010), disability is the main consequence of the concurrence of the aging process, lifestyles and health conditions [2].

In [1] (and compare also with [3] it is stated that the number of people aged 65+, in Europe, will almost double over the next 50 years, from 85 million in 2008 to 151 million in 2060. This is a great challenge for establishing new approaches with more efficient targets for public health and for older people. Hence, the aim is the increase of the life expectancy free of disability and therefore preventing and/or delaying the onset of dependence. This will favor optimization of opportunities for health, participation and security in order to improve quality of life as people age. That is active and healthy aging.

In the field of today’s data science there is a wide variety of new and sophisticated computational methods and also tools for building predictive models and performing enhanced data analysis. In clinical medicine these methods are used to offer support in tasks such as decision making based on the patient’s data. This covers the spectrum of diagnostic, therapeutic and monitoring tasks. Previous collected patient data can be used to build a predictive model which provides a prediction for the clinical outcome. Clinicians can act on this information and promptly react to possible or likely adverse events [4].

Such an adverse event is for example the onset of frailty, which in [5] is defined as a clinical geriatric syndrome (a more detailed explanation can be found in the subsection *Frailty* in *Related Work*). Frailty is characterized by a decreasing capacity to respond to demands of daily life, caused by diminishing functional reserve. The prevalence of frailty in people 65 + ranges from 7 to 16.3%, increasing with age, and it is the main risk factor for disability [6]. Therefore, frailty assessment is a key tool for the prevention of disability by identification of people at risk.

Data analytics can of course also be applied to analyze retrospective clinical data of the aging population which can be crudely separated into healthy and frail people. This, in order to help to find early predictors for frailty, which in turn would enable the creation of policies for early prevention and adequate early on treatment of the frailty syndrome. Furthermore, this may undoubtedly have a high beneficial impact on society. Sure enough this undertaking, in order to be fruitful, requires extensive medical records of elderly patients.

### Objectives

**The main aim of the present work is to demonstrate that data science applied to medical data of elderly, partly frail people can help to find new potential predictors and to obtain a predictive model for the frailty syndrome.**

In order to fulfill this goal we will focus on:

1. Building models that are able to discriminate between frail and non-frail people
2. Finding potential predictive factors for frailty

In fact we propose just to focus on frail subjects understood as any people either with the status pre-frail or frail according to the Fried scale [5].

## Related work

The main focus of this paper lies in building predictive models for the frailty syndrome and in discovering potential predictors. Consequently, it will be reviewed in what follows, the existing literature related to data mining in the medical domain and frailty.

### Data mining in the medical domain

Predictive data mining is becoming an important analytical instrument for the scientific community and clinical practitioners in the field of medicine [4]. Secondary use of patient and clinical study data is able to enhance health care experiences for individuals. Further, it enables the expansion of knowledge about diseases and treatments and leads to an increase of efficiency and effectiveness of health care systems [7]. Moreover, molecular data holds the potential to offer insights on single patients, therefore changing decision-making strategies. Thus, it seems predictive data mining will be a strong ally for the transformation of medicine from population-based to personalized practice.

Medical data has already successfully been used for developing various clinical decision support systems’ (CDSSs), which significantly impact practitioner’s performance and the health care process in a positive way and will do so in the future [8, 9]. Nevertheless, there still is a lot of room for improvement and the remaining issues have to be tackled.

Regarding building predictive models, the currently widely used neural networks (NN) [10] and also the deep learning approaches [11] are a very robust group of techniques with a good performance and they do deliver very promising results, but they are very hard to interpret because of their complex inner working. Simpler techniques like the naive Bayes classifier (NB) [12], linear discriminant analysis (LDA) [13], support vector machines (SVM) [14] and tree-based approaches [15] produce results that are much easier to interpret. Consequently, we propose in this paper to use the latter kind of techniques.

An important feature of the medical data due to its nature, is that in order to understand it, the involvement of the medical professional is paramount. Interactive machine learning (iML) [16] approaches allow to insert the physician in the “loop” of learning and that is what we have attempted to realize in this research.

### Frailty

The frailty syndrome was defined by Fried et al. [5] as a syndrome where three or more of the following criteria are present: unintentional weight loss (10 lbs/4.54 kg in the past year), self-reported exhaustion, weakness (measured via grip strength), slow walking speed, and low physical activity. Subjects with no deficits in all criteria score 0, which means they are not frail. Those who have deficits in 1 criterion or 2 criteria are called intermediate frail or pre-frail. All higher scores lead to the classification frail.

Frailty is considered highly prevalent in old age and associated with an elevated risk for falls, disability, institutionalization, hospitalization, and mortality [5]. However, it should not be considered synonymous with disability or comorbidity. Fried et al. state that comorbidity should rather be treated as an etiologic risk factor for frailty and disability as an outcome. Disability cannot be reversed, but it is preceded, sometimes by several years, by the frailty syndrome, which can be reversed, and thus prevented from worsening and its progression monitored.

Even that we use this work [5] as reference, in this research also other literature regarding frailty is presented. Apart from the index Fried et al. proposed, also others have emerged [17, 18]. Moreover, frailty is entangled with other concepts like disability and comorbidity and some effort has already been made to separate those [19]. Frailty has been also successfully used as a predictor itself, for example for predicting postoperative outcomes [20], where one study [21] found that it is more useful than conventional methods. These findings affirm the potential of the syndrome definitions and available indexes as being a stable concept.

Frailty seems to be strongly connected to physical activity and exercise, which have been proven to be protective factors [22, 23]. Further, it seems that the syndrome is closely related to mental impairment and mental health, especially depression [24]. Increased age and not having a daily consumption of vegetables and fruits were each associated with frailty or pre-frailty [25]. There is also a considerable gender aspect to this syndrome. Women are more likely to become frail in higher age and also frail women have a higher risk of developing disability, being hospitalized and death [26]. Moreover, some physiological blood parameters seem to be related to frailty and hold the potential to serve as markers and/or predictors. Studies found that this geriatric syndrome is also related to increased inflammation and elevated markers of blood clotting [27]. In a study done by Baylis et al. (2013) [28] the relationship between immune-endocrine parameters and frailty and also mortality after 10 years in females and males with an age between 65 and 70 years was investigated. Their findings were that higher baseline levels of white blood cell counts, lower levels of dehydroepiandrosterone sulfate (DHEAS) and higher cortisol to DHEAS ratio could be related to a higher probability of frailty in the future. Additionally, it was found that the presence of diabetes also is a risk factor for the onset of the frailty syndrome [25]. Concluding, a lot of suitable predictors (preventive and risk factors) have already been found and are used for frailty screening and also prediction.

From the previous review of literature related to the frailty syndrome the main conclusions are:

1. Fried’s frailty score [5] seems to be the one widely-used by physicians
2. In the research of Fried et al. the following factors are used to establish the frailty level (non-frail, pre-frail and frail):
  - unintentional weight loss (10 lbs in past year)
  - self-reported exhaustion
  - weakness (grip strength)
  - slow walking speed
  - low physical activity.
  These variables are highly correlated with the variable presenting the frailty status. Thus, we propose in our research to use any other factors (variables) to predict frailty.

## Methods

### Data

We used the data of the *Toledo Study for Healthy Aging* (TSHA). In [29] the TSHA is described as follows. The Toledo study is a population-based study conducted on 2488 individuals aged 65 years and older. The study subjects were selected by a two-stage random sampling from the Toledo region. Institutionalized as well as community dwelling persons were selected. Data was gathered in 3 waves: first (2006 to 2009) information on social support, activities of daily living, comorbidity, physical activity, quality of life, depression symptoms, and cognitive function was collected. Furthermore, anthropometric data (mass and length of body segments) and results of physical performance tests (walking speed, upper extremity and lower extremity strength, and the stand-and-sit from a chair test) were collected and a blood sample was obtained. Many of the used variables are also recommended by the American Geriatrics Society (AGS) for screening older patients for risk of falling and preventing falls. The diagnosis of the frailty syndrome was based on the Fried criteria (weakness, low speed, low physical activity, exhaustion, and weight loss)[5]. In the second wave (2011-2013) and in the third wave (2015-2017), which is ongoing, additional parameters were added (urine parameters). In the first wave the data of 474 patients was available, of which remained 354 in the second wave.

From the aforementioned Toledo study a subset of data has been made available for this work. In particular, anonymized data of 474 patients has been provided. Thereby, for each patient medical data consisting of 284 parameters was available. The majority of attributes belong to the first wave of the TSHA (2006-2009) and only 21 come from the second study wave conducted in 2011-2013.

#### Definition of the variables

The provided data set contains 284 variables from which only one is considered the variable to be predicted, the *FRAILTY* variable. Even that in the TSHA study this variable takes the values: non-frail, pre-frail and frail, for the present study the variable is a binary one in which the classes pre-frail and frail have been fused together. Therefore, the in this work used target variable *FRAILTY* consists of the classes *non-frail* (value: 0) and *frail* (value: 1).

Hence, in total 180 observations are *non-frail* and 294 observations are *frail*. The remaining 283 variables will be used to build models for the *FRAILTY* variable. They were grouped according to their semantics into: i) demographic, ii) phenotype, iii) medication and iv) code features. The phenotype features then were further split into physique, blood, cardiac, disease, self reported disease, consumption and medical test attributes.The medical test attributes were further divided into features corresponding to the Geriatric Depression Scale (GDS), Activities of Daily Living (ADL), Instrumental Activities of Daily Living (IADL), Mini-Mental-State-Examination (MMSE) and Mobility Scale (MS) attributes.

Below you can find a short explanation for each medical test, which was carried out in the TSHA [29]:

- **Geriatric depression scale (GDS)** This scale was created with the objective to obtain a reliable rating for depression in elderly. The applicant himself answers in the so called *short form* 15 different questions. Of those, 10 questions indicate the presence of depression when positively answered and the remaining 5 questions indicate the presence of depression when negatively answered. The test yields a score between 0 and 15, where scores between 0 and 5 mean no depression is present and values above 5 indicate the presence of a depression [30, 31].
- **Activities of daily living (ADL)** In this assessment also a questionnaire is used, which is answered by the patient. Here the goal is to estimate the patients’ satisfaction in his daily activities, which contain hygiene, alimentation and independent access to necessities. There exist different variations of the ADL test, which differ regarding their contained number of questions. In this work the ADL according to Katz [32] was used. The answers to 6 different questions provides a score between 0 and 6, where a score of 0 signifies no ability of self-care and a score of 6 complete ability of self-care.
- **Instrumental activities of daily living (IADL)** Like the ADL-test but mainly focused on instrumental activities. These include following daily tasks and responsibilities: food preparation, shopping, using the telephone, housekeeping, transportation, responsibility for own medications and the ability to handle finances. For each activity exist 3 to 5 questions, each yielding 0 or 1 point. The maximum for each category is 1 point and signifies that the ability to perform that certain task is given. At the end these points are summed up. This sum represents the IADL-Score with a range between 0 and 8. [33]
- **Mini-mental-state-examination (MMSE)** The Mini-Mental-State-Examination represents standardized test for cognitive function or measure of impaired thinking. The tested areas of cognitive function consist of orientation, registration, naming recall, calculation, writing, attention, repetition, comprehension, reading and drawing. The range of the result lies between total cognitive absence (0 points) and full cognitive function (30 points) [34, 35].
- **Mobility score (MS)** The MS questions belong to the Physical Activity Scale for the Elderly (PASE) questionnaire [36]. They provide validated knowledge about the physical activity of the patients. Here, 5 principal questions and follow-up questions were asked, yielding to a in this work derived score between 0 and 5. The maximum score indicates full mobility and 0 signifies extremely limited mobility.

### Data exploration and quality assessment

The retrieved data set was analyzed using different statistical visualization techniques like plotting the histogram, the kernel density function estimate and box-plots. Further, the values of each feature were inspected and compared to the values they should have according to the provided data dictionary (Additional file 1). Moreover, statistical measures were calculated and analyzed. The provided variables were divided according to their corresponding data type into continuous, categorical and binary variables. Depending on this data type, different visualizations were realized and statistical measures calculated. Features representing codes and IDs of the TSHA data do not contain relevant information with regard to frailty prediction, as they were created for organizational reasons and do not contain information regarding medical/phenotypic/demographic aspects.

In total the data set contains 474 observations and 284 features including the target variable representing the frailty status. In total 176 features are more than 90% complete and in 41 features more than 50% of the values are missing, of which 12 are follow up questions to a previous asked principal question. For example the feature *tab1* contains the answers to the question “Have you smoked at least 100 cigarettes in your entire life?”, when answered with 2 (which stands for no) the follow up question, represented by *tab1a* (“If yes, Did you smoke cigarettes daily, occasionally, or not at all?”), has not been asked. So as a matter of fact, these values are not missing at random, but rather the question was not applicable for these observations. One can see that in order to make use of all the observations and therefore of the contained information, a special strategy for dealing with missing data is clearly necessary. For many features a special treatment is necessary in order to better capture their actual meaning as the current values do not sufficiently reflect it.

Through analysis of known frailty related factors via ontology-guided PCA using the approach described by Wartner et al. (2016) [37], mining association rules using the apriori algorithm [38] and general correlation statistics, it can be assumed that the from the doctors described relationships are also present in the data.

### Data preparation

In this phase the data is cleaned, prepared and when necessary transformed. Further, new features are derived and the quality of the features in terms of predictiveness is assessed.

First the features are analyzed regarding their contained information in a statistical view and in a semantic view. In the scope of this work it was decided to exclude information regarding drugs. On the one hand because the information presented is not sufficiently structured and the pre-processing required exceeds the time available for this work and on the other hand, because doctors preferred to have the first predictive model only with phenotypical parameters and results of the different tests.

Features which belong to the follow-up study conducted in the years 2011-2013, were discarded, as there were only 21 of them (and the remaining 264 are from the earlier study wave) and therefore a temporal analysis was not possible. Also features, which in a statistical sense contain no information, were excluded. An example therefore is the feature that describes binarily the presence of leukemia or polycythemia. As all the observations have the same value “2” (meaning “not present”), this feature was excluded. Summing up, a total of 196 variables were left for further analysis.

The data set was inspected regarding potential outliers, using the reference variable ranges according to the data dictionary (Additional file 1). Not described appearing values were examined from a statistical point of view using the informal box plot method. Additionally, the kernel density estimate was analyzed. After that exploration, domain-knowledge was used to analyze the significance of appearing extreme values. Further, the doctors of the hospital were involved in the decision if the values are plausible and should be kept, or if they should be discarded. Moreover, possible/plausible values were discussed with them and thresholds were established, exceeding values then simply were set to not available (NA).

After the data had been cleaned, the following step was to extend the available data set by creating new features, using the available ones. Sometimes the doctors can provide some scores or ideas for building features. A simple example for this purpose is the Body Mass Index (BMI) [39], which can be easily calculated using the patients weight in kg and his height in cm (see Eq. 1).

1 $$  BMI = \frac {weight} {\left(\frac{height}{100}\right)^{2}}  $$

Other than the BMI also features representing the mobility score (sum of the answers to the principal questions of the mobility score, see subsection *Definition of the Variables*) and the total income (sum of household income and individual income) were created.

#### Imputation of missing data

Once data is prepared and new features have been derived, the following step is to make sure all the observations can be used in the modeling phase. Therefore, it was decided to calculate different estimates for each missing value. Thus, missing values are imputed (filled) with estimates.

In Table 1 the features where more than 5% of the values are missing can be seen. These measures are referring to the already in the previous steps pre-processed data set. An important step before applying imputation techniques, is to assess the reason for missingness. Three types of missing data exist and they are called Missing Completely At Random (MCAR), Missing At Random (MAR) and Missing Not At Random (MNAR). The assumed reason for the missingness and the according applicability of imputation techniques is also presented in Table 1. Features where more than one third of the values are missing were excluded from further investigations. They are marked in bold. Overall, all MNAR cases can be found in features which represent follow-up questions, they therefore were only be answered if the underlying basis question was answered positively. For them no imputation is possible because they can’t be derived from other features.

**Table 1:** Overview of features with more than 5% missing values

Feature	Percentage of missing data	Reason for missingness	Imputation possible
**Times stopped smoking**	**75.11**	MNAR (follow-up question)	No
**Daily wine consumption**	**91.14**	MNAR (follow-up question)	No
**Daily beer consumption**	**98.73**	MNAR (follow-up question)	No
**Daily spirits consumption**	**98.95**	MNAR (follow-up question)	No
**Duration of alcohol consumption**	**82.91**	MNAR (follow-up question)	No
Earlier alcohol consumption	19.20	MAR	Yes
**Kind of drinker (earlier)**	**86.29**	MNAR (follow-up question)	No
**Starting age alcohol consumption**	**86.50**	MNAR (follow-up question)	No
**Ending age alcohol consumption**	**86.92**	MNAR (follow-up question)	No
D Dimer [ *μ*g/L]	17.72	MAR	Yes
High-sensitivity C-reactive protein (hs-CRP) [mg/L]	14.98	MAR	Yes
Number of IADL abilities	6.33	MAR	Yes
Total MMSE score	15.82	MAR	Yes
Total GDS	9.49	MAR	Yes
Depression	9.49	related to *{*gdstotal}	No
Insulin [U/mL]	11.60	MAR	Yes
HDL	9.07	MAR	Yes
LDL	9.07	MAR	Yes
**Total testosterone [ng/dL]**	**37.97**	MAR	Yes
**Free testosterone [ng/dL]**	**37.97**	MAR	Yes
Mobility scale question 5	8.44	MNAR (follow-up question)	No
Mobility scale question 6	8.44	MNAR (follow-up question)	No
Mobility scale question 8	14.35	MNAR (follow-up question)	No
Mobility scale question 9	13.92	MNAR (follow-up question)	No
Mobility scale question 11	7.81	MNAR (follow-up question)	No
Mobility scale question 12	7.59	MNAR (follow-up question)	No
Mobility scale question 14	25.95	MNAR (follow-up question)	No
Mobility scale question 15	26.16	MNAR (follow-up question)	No
MMSE temporal domain 1	17.93	MAR	Yes
MMSE temporal domain 2	18.78	MAR	Yes
MMSE temporal domain 3	18.14	MAR	Yes
MMSE temporal domain 4	22.57	MAR	Yes
MMSE temporal domain 5	12.87	MAR	Yes
MMSE spatial domain 1	13.08	MAR	Yes
MMSE spatial domain 2	13.29	MAR	Yes
MMSE spatial domain 3	13.29	MAR	Yes
MMSE spatial domain 4	13.29	MAR	Yes
MMSE spatial domain 5	13.29	MAR	Yes
MMSE remembering 1	18.99	MAR	Yes
MMSE remembering 2	19.41	MAR	Yes
**MMSE backward counting**	**51.05**	MAR	Yes
**MMSE spell the word**	**61.60**	MAR	Yes
MMSE object naming	13.92	MAR	Yes
MMSE repeat phrase	13.08	MAR	Yes
MMSE left right	13.50	MAR	Yes
MMSE following written order	13.29	MAR	Yes
MMSE write sentence	13.92	MAR	Yes
MMSE copying design	13.50	MAR	Yes
Cognitive impairment	17.09	MAR	Yes
Individual income	8.44	MAR	Yes
Household income	13.29	MAR	Yes
Number of persons in the family	18.78	MAR	Yes
Insulin like growth factor 1 (IGF1) [ng/mL]	27.00	MAR	Yes
**Dementia type**	**98.73**	MNAR (follow-up question)	No
Overall income	13.71	MAR	Yes

Features where more than one third of the values are missing are presented in bold

In order to use all the available information contained in the data set, different imputation settings using the MICE implementation, more specifically the *CALIBERrfimpute* [40] expansion of it, were considered. Following configuration, regarding the imputation method, was chosen:

- For continuous features: **rfcont** for numeric random forest (RF) imputations
- For binary, ordered and unordered categorical features: **rfcat** for categorical RF imputations (factor, ≥ 2 levels)

Due to the size of the data set and the high number of features it was decided to use a selection of suitable features for the imputation models. One way is selecting manually every predictor for every imputation model and another way is to use statistical measures for the selection. Consequently, is it for example possible to just consider variables which show a correlation higher than a certain specified percentage. As it is a rule to use as much information as possible as this leads to multiple imputations which have a minimal bias and a maximal certainty [41], a minimal correlation-threshold of 7% was used.

Additionally, only such variables which are more than a certain desired percentage complete will be used. For the first imputation only predictors, which correlate more than 7% and are more than 80% complete were selected by configuring the parameter *pred*. The overall configuration of the *mice()* function can be seen in following code-fragment.


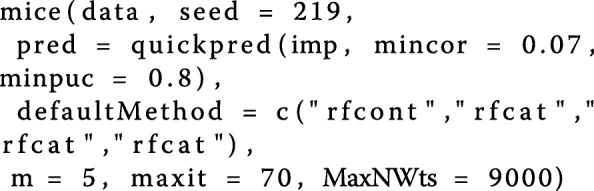


Here, *MaxNWts* depicts the maximal number of weights used by the inner neural network. The argument *maxit* was used to set the maximal numbers of iterations to 70. As creating 5 different imputations was desired, the parameter *m* was set to 5. The argument *defaultMethod* contains the different per default used methods for the different data types, which were already mentioned earlier. Using *pred*, different restrictions regarding minimum correlation and completeness of the predictors were added. The first argument represents the data set in matrix form for which the imputations should be computed. The parameter *seed* can be used to set the number for initializing the pseudo-random generator.

The mean and the standard deviation for each variable at each iteration can be observed in the received imputation object. These values were plotted for the features with the highest amount of missing values in order to see if median and variance of the different imputations do converge. It seemed that 70 iterations are quite sufficient in this regard.

The obtained imputations are then examined using visualization tools. One possibility to check if the obtained imputations are reasonable, is to compare the kernel density estimates of the observed and the imputed values for ideally all variables. As this would not have been feasible within the scope of this work, only features with more than 5% missing values were examined. Further, the kernel-density function was plotted and analyzed for each feature and each imputation in order to evaluate the quality.

**Fig. 1:**
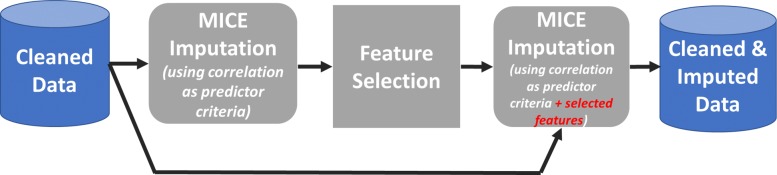
Imputation Process. This figure illustrates how the imputation and the feature ranking process are connected. At first, the imputation models are built using features, which show a minimum correlation (here 7% was used) to the feature to be imputed. After that, the obtained 5 different data sets are used for the feature selection process. Knowing the selected features, the imputation is re-done. This by using as predictors additional to the correlated features also the selected ones

The second imputation was done the same way, but this time also the selected features were included for every imputation model. This is recommended by Buuren and Groothuis-Oudshoorn (2011) [41]. The connection between the imputation and the feature selection process is demonstrated in Fig. 1.

The overall configuration of the *mice()* function for the second imputation can be seen in following code-fragment.


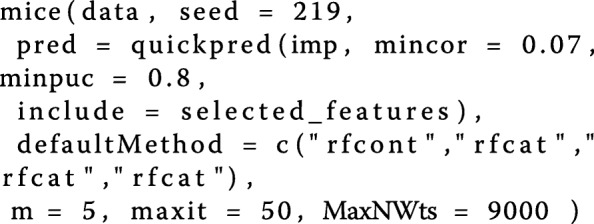


The only difference is that by adding the parameter *include* =*selected_features* to the attribute *pred*, the selected features are used additionally for every imputation model.

Here, the obtained imputations were also analyzed as it has been done before. With the help of density plots of the imputed and the original values, once again the quality of the imputations was assessed. The obtained 5 different imputed data sets then were used for the modeling process.

### Feature selection

As the objective is to predict the *FRAILTY* variable with a subset of features, which are highly predictive, the most predictive features were determined using feature ranking methods. Further, the obtained results were compared with the suggested factors from the doctors of the Toledo study.

In order to make just use of the features which are indeed predictive and therefore beneficial for the final predictive model in terms of performance, different feature selection methods were considered. Finally, it was decided to use the *Boruta* [42] algorithm, which uses a RF wrapper method. The implementation of the R package *Boruta* [42] was used. The selection was performed with regard to the binary target variable *FRAILTY*.

**Fig. 2:**
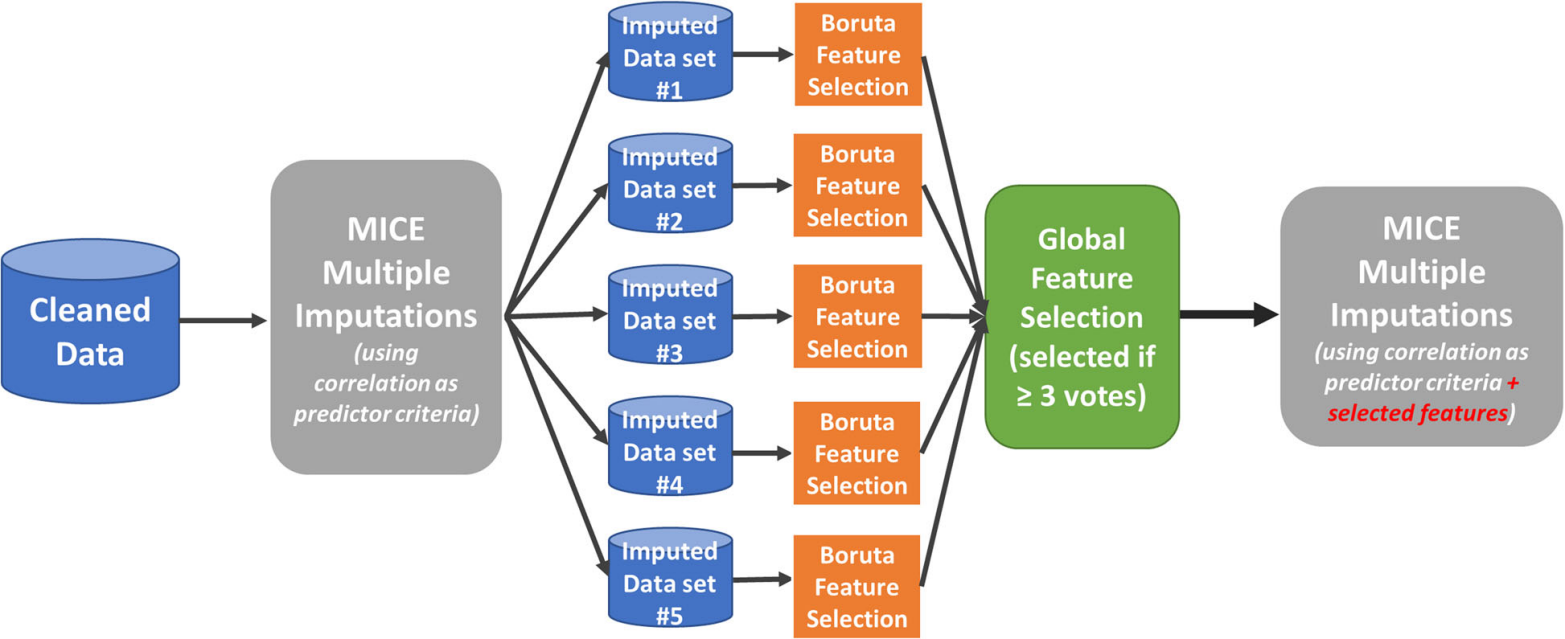
Feature Selection Process. This figure shows the overall feature selection process. At first, the *Boruta* algorithm is applied on each imputed data set. Then, the 5 different selected feature sets are compared and features which appear in 3 or more selected sets are chosen for the final feature set

For each imputed data set the feature selection process using the *Boruta* algorithm was executed. For the sake of obtaining reliable and stable results, the method was configured to use 1000 trees for the RF algorithm and to perform 1000 runs in order to avoid so called tentative results. This means there are still features, which could not be rejected nor accepted for the final set. At the end, 5 different sets of selected features were present. The finally chosen selected features were those, which appeared at least 3 times in the 5 different *Boruta* sets. The complete feature selection process, which begins after the first executed imputation procedure and provides the selected features for the second imputation, is shown in Fig. 2.

**Fig. 3:**
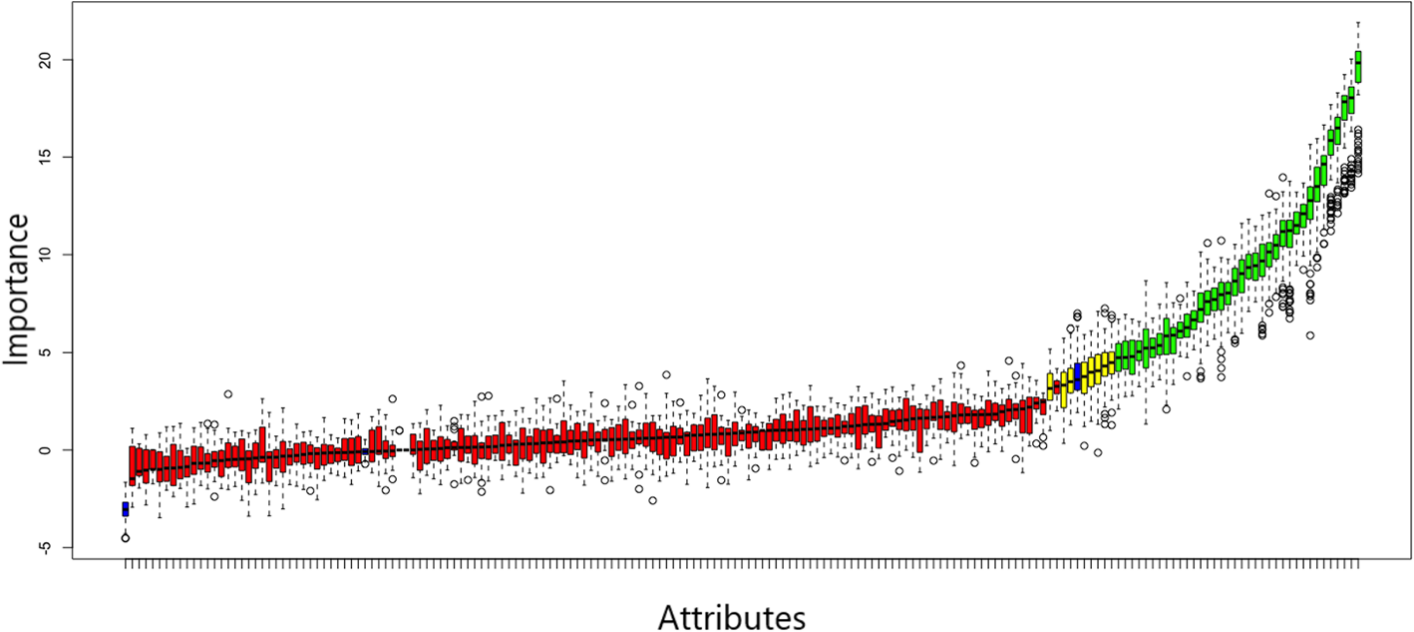
Feature Selection Results (Boruta). This image shows the attributes and their importance measure, by which they were selected (green) or rejected (red). This decision was made by comparing their importance measure to randomly permuted copies of themselves, the so called shadow attributes [54]. Features which could neither be selected nor rejected were marked tentative (yellow)

**Findings** In Fig. 3 the result of the feature selection is presented. The variables are ordered by importance, the rejected ones are colored red, the selected ones green and those, for which no decision could be made, are yellow. All the importance measures of the features were compared to randomly permuted copies of themselves, so called shadow attributes. The Z-Score of the most important shadow attribute was used as separator between selected and rejected features. Features where no decision could be made were marked tentative and colored yellow.

By using the function *TentativeRoughFix* those features, with a median importance higher than the maximal one of the shadow attributes, were selected and the others rejected. This is a simple test for judging these tentative attributes. Tentative attributes could also be resolved by increasing the number of importance runs of the *Boruta* algorithm. That is why instead of the default 100 runs, 1000 runs were used.

After the feature selection, the obtained final variables were used for another imputation round. As suggested by Buuren and Groothuis-Oudshoorn (2011) [41], the features which are powerful in terms of predictiveness, with regard to the target variable, should always be used in the imputation for each feature. That is why they all were included in each imputation model.

### Modeling and evaluation

Once data had been prepared, the following step was to build predictive models. As can be seen in the sections to come, different techniques have been applied. Later the received results have been compared and validated. In what follows, one can find the model settings, the modeling and validation schema, the model performance and lastly the evaluation of the models.

#### Classification model settings

Following learning algorithms for the predictive models have been chosen: the NB algorithm, Classification And Regression Trees (CART), bagging CART, C5.0, RF, SVM and LDA. They have been selected as representative of the most widely used in the literature [43]. This variety of algorithms allows to analyze the robustness of the solution and how different methods influence the performance. The different algorithms were implemented in the R environment using different third party packages, which are listed in what follows. Further, changed configurations, which differ from the default settings are described in this listing.

##### NB

The NB classifier *naiveBayes* of the R package *e1071* was used in its standard configuration.

##### CART

The CART algorithm *tree* of the same titled R package was used in it’s standard configuration.

##### Bagging CART

The bagging CART implementation *bagging* from the R package *ipred* lead to the best results, when using 55 bootstrap replications.

##### C5.0

The best accuracy for the C5.0 algorithm (from the R package *C50*) could be achieved using 50 iterations for the multiclass classification and 55 iterations for the binary classification.

##### RF

The best accuracy in the RF implementation “randomForest” from the R package with the same name was achieved, using 1000 trees, no replacements in the inner sampling of cases and 5 as number of variables randomly sampled as candidates at each split.

##### SVM

The best setting for this algorithm was using as type the C-classification, as kernel the radial basis function (RBF) and as tolerance of termination criterion the value 10^−3^. The degree was set to 3, the ’C’-constant of the regularization term in the Lagrange formulation was set to 10 and the gamma of the RBF was set to 0.07.

##### LDA

This method from the R package *MASS* was used in its standard configuration.

#### Optimization of algorithm input

In order to utilize the data in the best way, it has been shown that sometimes it is beneficial for the performance of the learning algorithms to transform the data to different ranges and also to change the distribution. This was also considered in this work and therefore, every algorithm was used on the z-score standardized, the Min-Max normalized and the raw data set. Where the raw form represents the data after completion of the preprocessing phases.

##### Min-Max Normalization

Min-Max normalization is a method where the values of the data are transferred into a range of [0,1]. Where the lowest appearing value *x*_*min*_ is set to zero and the maximal value *x*_*max*_ is set to 1. The used formula is shown in Eq. 2. Here each value *x*_*i*_ is Min-Max normalized using its current value, *x*_*min*_ and *x*_*max*_.

2 $$ mm(x_{i}) = \frac{x_{i} - x_{min}}{x_{max} - x_{min}}  $$

For each learning algorithm the 3 aforementioned input data set variants were used and the resulting performances were compared. Then for each algorithm the variant which leads to the best performance was chosen.

### Modeling and validation schema

**Fig. 4:**
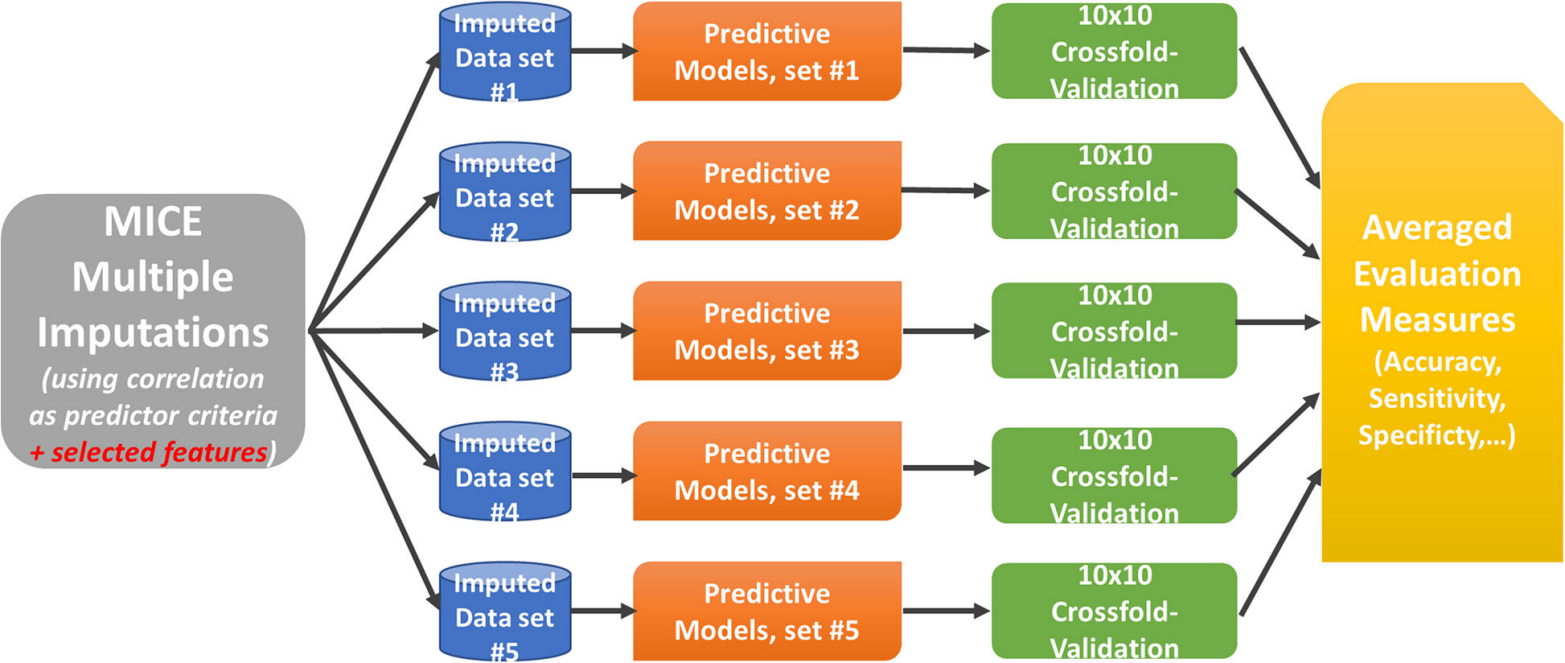
Modeling and Evaluation Procedure. This image shows the general modeling and evaluation procedure. Firstly, models are built using the 5 different obtained imputed data sets. Secondly, the models are evaluated in a cross-fold validation setup. Then the resulting performance measure values (e.g. accuracy, sensitivity, specificity) can be compared

After preparing the data for the modeling phase, the next step was building the models and validating them. In Fig. 4 the procedure for modeling and evaluating is presented. At the beginning each obtained imputed data set is used to build the different models (e.g. RF, DT, SVM), which are tested in a cross-fold validation setup. The resulting performance measure values of each model for each imputation are then compared and the one with the overall best performance is chosen as final model. Therefore, 5 different final models are obtained at the end. Afterwards they can be used as an ensemble classifier, which provides one result for new unseen instances.

In order to evaluate the out of sample error of the built models, as mentioned before, the very commonly used 10-fold cross-validation was performed. Here, the training data is split into 10 different, generally equal-sized folds. Then, for each fold *k* the model is trained on all the folds but the *k*’th. After that the obtained model is tested on the *k*’th fold. This is repeated for all 10 folds, where *k*=1…10. The error averaged over all the folds is then computed.

Due to the fact that the classes are imbalanced (180 observations are *non-frail* and 294 observations are *frail*), a stratification technique was implemented. This in order to have the same number of *frail* and *non-frail* observations in each created fold and thereby maintaining the initial class balance. At first, the observations were split according to their frailty status (2 classes). Afterwards, the 10 folds were created separately for each class and then fused according to the fold-number. The observations were chosen randomly.

By using multiple 10-fold cross-validations, a first estimate of the generalization error is obtained. For the modeling phase five different imputed data sets were considered, thereby five different best performing classifiers were obtained. The final predictive model represents an ensemble classifier, which can be used on new unseen instances. The final predicted class is the result of five different votes, where each vote is the corresponding classification result of each model.

#### Performance measures

The confusion matrix of the prediction results is the basis for the following used measures: accuracy, sensitivity (also called true positive rate), specificity (also called true negative rate), precision, *F*_1_-Score and the area under the receiver operating characteristic curve (AUC) [44]. A more detailed description of the used performance measures can be found in [45].

## Results

### Selected features

The finally selected features, which appeared at least 3 times in the 5 different feature selection sets, using the *Boruta* algorithm, can be seen in Table 2.

**Table 2:** Obtained final selection of features using the Boruta algorithm and a voting system (presence of the feature in at least 3 out of the 5 sets)

Description	Type
Height (cm)	Numeric
Presence of cognitive impairment	Binary
Presence of depression	Binary
Mobility Scale follow-up question (tiredness when going out)	Binary
Mobility Scale question (stair-climbing ability)	Binary
Mobility Scale follow-up question (tiredness when walking outside)	Binary
Mobility Scale question (walking outside ability)	Binary
MMSE follow-up question (remembering objects ability)	Categorical
Total GDS	Binary
Age in years	Numeric
ADL question (difficulty washing)	Categorical
Number of ADL abilities	Numeric
Number of IADL abilities	Numeric
IADL question (difficulty using telephone)	Categorical
IADL question (difficulty shopping)	Categorical
IADL question (difficulty cooking)	Categorical
IADL question (difficulty doing light housework)	Categorical
IADL question (difficulty doing heavy housework)	Categorical
IADL question (difficulty using public transportation)	Categorical
Total MMSE score	Numeric
Sum of mobility score main features (em1,em2, em3,em4,em5)	Numeric
Number of drugs (drug intake)	Numeric
Alkaline phosphatase [U/L]	Numeric
Presence of polypharmacy	Binary
Self-reported health status	Categorical
Self-reported health status compared to people the same age	Categorical
Capacity of dealing with problems	Categorical
Capacity of dealing with tasks	Categorical
GDS question (dropped activity of interests)	Binary
GDS question (boredom)	Binary
Presence of joint inflammation (more than 4 weeks in a row)	Categorical

### Model performance

The model performances were obtained by averaging each performance measure for the 10 different 10-fold cross-validation setups. The obtained results can be seen in Table 3. For each performance measure, the over the folds averaged value including the standard deviation is shown. The highest obtained value for each performance category is marked in bold.

**Table 3:** 10-fold cross-validation results for the binary classification models for each imputed data set, working with the two classes *non*−*frail* and *frail*

Prediction method	Accuracy	AUC	Sensitivity	Specificity	Precision	F_1_-Score
Imputation 1						
Naive Bayes	73.20 ± 5.97%	0.756 ± 0.052	0.656 ± 0.102	**0.856** ± **0.079**	**0.885** ± **0.054**	0.749 ± 0.067
CART	72.77 ± 5.20%	0.710 ± 0.061	0.782 ± 0.108	0.639 ± 0.168	0.789 ± 0.065	0.778 ± 0.049
Bagging CART	75.51 ± 7.16%	0.731 ± 0.070	0.830 ± 0.086	0.633 ± 0.084	0.786 ± 0.048	0.806 ± 0.060
C5.0	**77.83** ± **7.13%**	0.752 ± 0.086	**0.860** ± **0.056**	0.644 ± 0.164	0.804 ± 0.075	**0.829** ± **0.051**
Random forest	77.64 ± 5.62%	0.755 ± 0.053	0.844 ± 0.089	0.667 ± 0.087	0.806 ± 0.041	0.823 ± 0.050
Support vector machines (RBF)	77.64 ± 6.55%	**0.762** ± **0.065**	0.824 ± 0.09	0.700 ± 0.099	0.819 ± 0.053	0.819 ± 0.057
Linear discriminant analysis	75.11 ± 5.34%	0.739 ± 0.042	0.789 ± 0.096	0.689 ± 0.047	0.805 ± 0.023	0.795 ± 0.055
Imputation 2						
Naive Bayes	72.78 ± 6.47%	0.750 ± 0.059	0.656 ± 0.109	**0.844** ± **0.094**	**0.878** ± **0.063**	0.745 ± 0.072
CART	70.89 ± 5.94%	0.699 ± 0.057	0.741 ± 0.098	0.656 ± 0.104	0.781 ± 0.047	0.757 ± 0.058
Bagging CART	75.11 ± 6.59%	0.729 ± 0.072	0.820 ± 0.089	0.639 ± 0.134	0.792 ± 0.066	0.802 ± 0.054
C5.0	77.39 ± 7.35%	0.745 ± 0.093	**0.867** ± **0.057**	0.622 ± 0.192	0.797 ± 0.082	**0.828** ± **0.050**
Random forest	77.01 ± 6.65%	0.752 ± 0.064	0.827 ± 0.101	0.678 ± 0.101	0.809 ± 0.052	0.815 ± 0.060
Support vector machines (RBF)	**77.63** ± **7.01%**	**0.761** ± **0.071**	0.827 ± 0.085	0.694 ± 0.102	0.816 ± 0.057	0.820 ± 0.060
Linear discriminant analysis	76.14 ± 5.15%	0.752 ± 0.046	0.792 ± 0.081	0.711 ± 0.057	0.817 ± 0.032	0.803 ± 0.050
Imputation 3						
Naive Bayes	73.41 ± 5.64%	0.757 ± 0.057	0.664 ± 0.083	**0.849** ± **0.102**	**0.885** ± **0.069**	0.755 ± 0.056
CART	73.21 ± 5.75%	0.728 ± 0.07	0.746 ± 0.064	0.709 ± 0.14	0.815 ± 0.067	0.776 ± 0.045
Bagging CART	78.28 ± 3.92%	0.764 ± 0.057	**0.841** ± **0.058**	0.688 ± 0.148	0.823 ± 0.062	0.828 ± 0.026
C5.0	74.06 ± 7.12%	0.709 ± 0.089	0.837 ± 0.057	0.581 ± 0.181	0.774 ± 0.073	0.802 ± 0.048
Random forest	77.62 ± 6.65%	0.762 ± 0.076	0.820 ± 0.068	0.704 ± 0.134	0.824 ± 0.068	0.820 ± 0.052
Support vector machines (RBF)	**79.32** ± **5.00%**	**0.779** ± **0.056**	0.838 ± 0.049	0.720 ± 0.09	0.833 ± 0.048	**0.834** ± **0.040**
Linear discriminant analysis	78.47 ± 4.77%	0.773 ± 0.051	0.821 ± 0.059	0.726 ± 0.085	0.833 ± 0.045	0.825 ± 0.040
Imputation 4						
Naive Bayes	72.78 ± 5.89%	0.750 ± 0.061	0.657 ± 0.083	**0.843** ± **0.111**	**0.881** ± **0.075**	0.749 ± 0.057
CART	71.26 ± 5.83%	0.697 ± 0.053	0.762 ± 0.095	0.631 ± 0.083	0.774 ± 0.043	0.765 ± 0.058
Bagging CART	76.38 ± 5.77%	0.747 ± 0.069	0.817 ± 0.076	0.676 ± 0.147	0.812 ± 0.065	0.811 ± 0.046
C5.0	74.25 ± 7.13%	0.712 ± 0.085	**0.837** ± **0.057**	0.587 ± 0.157	0.774 ± 0.07	0.803 ± 0.052
Random forest	76.99 ± 5.90%	0.755 ± 0.069	0.817 ± 0.069	0.693 ± 0.136	0.819 ± 0.067	0.815 ± 0.046
Support vector machines (RBF)	**78.47** ± **5.14%**	0.771 ± 0.057	0.827 ± 0.053	0.714 ± 0.092	0.829 ± 0.049	**0.827** ± **0.041**
Linear discriminant analysis	78.06 ± 5.39%	**0.772** ± **0.057**	0.807 ± 0.061	0.737 ± 0.091	0.837 ± 0.049	0.820 ± 0.045
Imputation 5						
Naive Bayes	73.41 ± 5.45%	0.756 ± 0.053	0.664 ± 0.088	**0.849** ± **0.098**	**0.885** ± **0.066**	0.754 ± 0.057
CART	71.67 ± 7.79%	0.702 ± 0.087	0.762 ± 0.100	0.642 ± 0.166	0.786 ± 0.089	0.769 ± 0.066
Bagging CART	76.79 ± 4.69%	0.749 ± 0.053	0.827 ± 0.071	0.671 ± 0.115	0.809 ± 0.049	0.815 ± 0.039
C5.0	75.31 ± 4.08%	0.726 ± 0.055	**0.837** ± **0.065**	0.615 ± 0.138	0.787 ± 0.055	0.808 ± 0.030
Random forest	78.03 ± 5.10%	0.764 ± 0.060	0.830 ± 0.073	0.698 ± 0.129	0.824 ± 0.061	0.824 ± 0.041
Support vector machines (RBF)	**78.47** ± **5.39%**	**0.771** ± **0.059**	0.827 ± 0.055	0.714 ± 0.092	0.828 ± 0.049	**0.827** ± **0.043**
Linear discriminant analysis	77.62 ± 5.35%	0.769 ± 0.058	0.800 ± 0.063	0.737 ± 0.102	0.836 ± 0.054	0.816 ± 0.045

The highest obtained value for each performance category for each imputed data set is marked in bold

## Discussion

The goal of this work was to build models that are able to discriminate between frail and non-frail people and to find potential predictive factors for frailty using data mining. For this purpose the medical data provided from the TSHA was used.

### Data understanding

The data understanding phase has shown to be useful to understand the relationship between variables and to find outliers, correlations and obtain general insights that have guided later the predictive modeling process. In fact the analysis of all the features helped to determine their particular importance in the frailty prediction. Further, the application of the ontology-based PCA approach described by Wartner et al. (2016) [37] was able to deliver some insights, which were further investigated.

### Selected features

A step of the process that resulted to be especially important was feature selection, given the high number of variables that were present. Using a RF wrapper based feature selection method, potential predictors were identified. Further, previously known predictors for frailty, from the medical community, could be used to validate the built model and vice versa, the feature selection process confirmed their predictability. The present work has identified potential predictors for predicting frailty, which were conformed by the doctors. Most of the found predictors are variables describing the mobility, the mental state and the capability of performing daily tasks.

Some interesting findings, according to the physicians is for example the presence of blood alkaline phosphatase level in *U/L* in the selected feature set (Table 2). Less surprising is that age is also among these features. Moreover, the final feature set also included variables regarding: depression (presence of depression, total GDS, 2 ^*nd*^ GDS question, 4^*th*^ GDS question), polypharmacy (presence of polypharmacy, number of drugs), mobility (mobility score), Mini-Mental-State-Examination (total MMSE score, presence of cognitive impairment, MMSE follow-up question [remembering objects ability]), Instrumental Activities of Daily Living (number of IADL abilities and the first 6 IADL questions), Activities of Daily Living (number of ADL abilities and ADL question [difficulty washing]), self-reported health-status (self-reported health status, self-reported health status compared to people the same age, capacity of dealing with problems, capacity of dealing with tasks) and rheumatic disease (presence of joint inflammation [more than 4 weeks in a row]), which also according to the doctors seem to be relevant.

The found feature set seems to be consistent with known frailty risk factors or preventive factors found by the medical community. Interesting seems to be the finding that the feature *p*40*falc*, representing the blood alkaline phosphatase level in U/L, is highly predictive. This certainly requires some follow up investigations, as this could possibly be a new biomarker for frailty detection. The doctors said that this variable is probably a good predictor, because it gives information about inflammation processes in the body. They are already investigating it, in the scope of the FRAILOMIC initiative [46], which is a research project aiming to identify the factors that turn frailty into disability. The doctors conformed that the found predictors are related to frailty. They commented also on the missingness of the gender feature. According to them, it’s one of the important markers for determining frailty and they were surprised that it did not appear in the final predictor set. It is possible that the feature selection algorithm found this variable to be redundant and that the contained information is already provided by other features. The variable height is, for example, highly correlated to the gender variable (correlation coefficient = 0.725). This manifests that further analysis with a bigger population is required in order to understand the role of this variable in particular but also for all the found potential predictors.

The set presented, results to be the best subset of features for the task of predicting frailty, even that some of these variables showed to be correlated in the data understanding phase. Machine learning algorithms are very flexible with regard to problems of multicollinearity, especially tree based ones and the SVM [47, 48]. In fact, that are the methods which were used for the predictive modeling. Consequently, the possible collinearity impact on the prediction models is avoided.

### Evaluation of the built models

For this research two different evaluations are required. First, the analyses of the performances of the models and later, the analysis of how the models actually fit the goals. The overall best performances in nearly all measures have SVMs with a RBF as kernel. Followed by RF, LDA, bagging CART, C5.0, NB and CART. Striking is the high obtained specificity and precision of the NB classifier, while it performs inferior in the other measures compared to the other models. In this case specificity represents the ratio of predicted real non-frail patients to all non-frail patients. Thus, this classifier shows an extraordinary performance in the task of detecting non-frail patients. The highest values for accuracy and AUC are always achieved by RF and SVMs, which do not differ significantly in their results. The highest scores in each category for each imputation are marked in bold in Table 3. The variation of the results between the different imputed data sets is also very small, which indicates that also the variation of the imputed values is small. For example, the accuracy of SVM averaged over all imputed data sets is 78.31 ± 0.70%. The standard deviation is not even one percent. The RF algorithm performed slightly inferior with an averaged accuracy of 77.46 ± 0.45%. Here the standard deviation is below a half percent, which shows that the performance is quite stable.

The built models achieved an accuracy of more than 78% for binary classification of the frailty variable, without using features, which are directly related to the target or used to build it (see Fried’s frailty criteria and stages [5]). The results show, that it is feasible to build predictive models for the frailty syndrome using medical data.

### Interpretability of the built models

The tree models derived by CART and C5.0 are easy to interpret as they can provide “human-friendly” explanations, which is an extremely important aspect (see e.g. [49]). Tree structures are ideal for capturing interactions between the features and present themselves a natural visualization with edges and nodes. Hence, good explanations could be derived, although linear relationships are presented by splits. When bagging is used (bagging CART, RF), the resulting model is not a single tree but an ensemble of trees, which significantly decreases the interpretability. NB is a simple and interpretable model. The contribution of each feature towards the final chosen class is clear. LDA also provides a result, which is very easy to interpret, as the output is a linear combination of the features. In case of the SVM the interpretability depends on the chosen kernel. If a non linear kernel is used, as in this work, the relationships can not be easily captured.

### Limitations

Predictive models, using the predictors obtained in the feature selection process, were built in order to predict frailty in patients. It was decided to derive a binary classifier, which is able to separate the two classes *non-frail* and *frail*. The classes *pre-frail* and *frail* from the original multiclass problem were fused into the class *frail* in order to work on a binary classification problem. Even though that could cause a degradation of the performance of the built models.

Further, it has to be stated that the derived model is technically speaking a predictive model, but presents semantically a diagnostic model. As already mentioned, temporal analysis was not possible as mainly data from one point in time was available. Thus, the built model relies only on data which has been collected at the same time as the diagnosis has been made.

By using multiple 10-fold cross-validations, a first estimate of the generalization error is obtained. Though, according to Bellazzi et al. (2011) [50] the prediction performance should also be tested on an independent data test set from another study.

Regarding the performed imputations of missing values, one could argue that using correlation between variables as a criteria for the imputation process could boost existing correlations, however multiple imputations have been used to reflect the degree of uncertainty when making use of such an approach and therefore not an exact result but an performance estimate has been presented.

Null imputation is a task that on its own requires a lot of work due to the vast amount of decisions that have to be made. In fact for each attribute a deep analysis is required. In this work 157 attributes are given for which data imputation is required. Due to the fact that the main goal of the present work is showing that prediction of frailty is feasible rather than analyzing the most efficient algorithm for a prediction, quite enough effort has been dedicated to null imputation. However, a deeper analysis would be needed in order to answer questions related to the statistical analysis of the multiple imputations and also to the obtained statistical results, which are pooled into a final point estimate plus standard error, applying Rubin’s pooling rules [51].

It is also important noting that several issues make medical data mining a hard task today. On the one hand, problems related to legal issues and all the issues concerning privacy and confidentiality and on the other hand, the problem of interoperability of systems make it difficult to have a complete view of the patient or to integrate data from different services at the hospital. Besides, one cannot forget the effort of obtaining a complete cohort of patients from which we can extract results. Consequently, in this work we would only analyze a cohort of 474 patients for which 284 variables were available. These data limitations allowed for only rough performance estimates for the models. It would be desirable to have a bigger sample, so that results would become more significant and validations would be possible in different cohorts.

## Conclusions

In this paper the feasibility of applying data mining techniques in order to extract models for frailty prediction using medical data from patients some of which are frail, has been analyzed.

From the work developed, it has been shown that in fact it is possible to extract meaningful patterns. Further, the importance of data preparation and data understanding for the successful extraction of predictive patterns has been demonstrated. Despite the importance of intelligent algorithms to extract the patterns, in this work we have additionally shown the paramount importance of pre-processing. Without a modest amount of effort in this phase, a reliable prediction model can not be built. Therefore, investing a lot of work here proved to be highly beneficial in terms of accuracy and reliability of the obtained predictions.

## Future work

This work contributed towards obtaining predictive models that can anticipate the onset of age related deterioration. In particular, the problem of frailty has been analyzed in this paper. However, for these models to be used in daily routine, some work still needs to be done, nevertheless, this work opens new lines of research.

A next step is to analyze the best algorithm depending on the size of the data set. In this work the main focus was to show that data analysis is possible rather than showing which methods are the most efficient. Consequently, in future work the feature selection process should be repeated once data of more patients is available. Moreover, we have focused on obtaining models for a binary variable *FRAILTY*, but in the future more models should be created in order to analyze differences between the the stages non-frail and pre-frail, and pre-frail and frail respectively.

All in all, one remaining task is removing step by step the expert from the deep processes of the data preparation pipeline by further developing the autonomy of the system. Yet, according to Holzinger (2017) [52] it seems to be unrealistic that such fully automatic approaches can be realized in the near future, most of all it is very important in the medical domain to foster transparency and trust. Standard black-box approaches lack transparency, hence do not foster trust and acceptance. Rising legal and privacy aspects, e.g. with the new European General Data Protection Regulations will make it more important in the future to explain why a decision has been made [53]. So in contrast to pursue the objective of increasing the autonomy of the process, it should be considered to include doctors as agents in the development of the predictive algorithm, in order make their domain-knowledge during the learning process available, which potentially could increase the performance of the final model [52]. Maybe in a more distant future fully automatic approaches will be feasible.

Besides, future research will include analyzing more features. In particular investigating the impact of medication or the impact of nutritional information is very promising. Moreover, future work could also focus on evolution analysis of patients regarding the frailty syndrome. The most important future work is to validate the results in other cohorts and it is necessary to check how the model

Albeit the results seem to be very promising, for them to have more impact, it would be required to further validate the results in other cohorts, along with testing on how the derived model from retrospective data performs in prospective trials.

## Additional file

Additional file 1 Data Dictionary: Description of the Variables. This file “Additional File 1.pdf” contains tables where for each available variable the name, expected values, a description and the data type is stated. The variables where divided into semantic groups, where for each group a table has been created. These groups have already been mentioned in the subsection *Definition of the Variables*. (PDF 93 kb)

## Abbreviations

ADL: Activities of daily living
AUC: Area under the curve
CART: Classification and regression trees
CDSS: Clinical decision support system
DALY: Disability adjusted life year
DHEAS: Dehydroepiandrosterone sulfate
GDS: Geriatric depression scale
IADL: Instrumental activities of daily living
iML: Interactive machine learning
LDA: Linear discriminant analysis
MAR: Missing at random
MCAR: Missing completely at random
MMSE: Mini-mental-state-examination
MNAR: Missing not at random
MS: Mobility scale
NA: Not available
NB: Naïve Bayes
NN: Neural network
PASE: Physical activity scale for the elderly
RBF: Radial basis function
RF: Random forest
SVM: Support vector machine
TSHA: Toledo study for healthy aging
