# Supplementary material for: Importance of medical data preprocessing in predictive modeling and risk factor discovery for the frailty syndrome
Source: BMC Med Inform Decis Mak. 2019 Feb 18;19:33. doi: 10.1186/s12911-019-0747-6 (PMC6483150; doi:10.1186/s12911-019-0747-6)
Supplement: Supplementary file 1 — Data Dictionary: Description of the Variables. This file “Additional File 1.pdf” contains tables where for each available variable the name, expected values, a description and the data type is stated. The variables where divided into semantic groups, where for each group a table has been created. These groups have already been mentioned in the subsection Definition of the Variables. (PDF 93 kb) [file 12911_2019_747_MOESM1_ESM.pdf]

Additional File 1

A.1 Tables

**Attribute name**	**Values expected**	**Description**	**Type**	**How and when was it recorded?**
ppeso	0,1	Fried criterium: weight loss >10 lbs. in past yr	categorical	calculated by hospital 2008
exhaustion	0,1	Fried criterium: exhaustion >=3days in past week	categorical	calculated by hospital 2008
pasefrag	0,1	Fried criterium: PASE <=20 percentile	categorical	calculated by hospital 2008
marchafragil	0,1	Fried criterium: time to walk >=80th percentile	categorical	calculated by hospital 2008
fuerzafragil	0,1	Fried criterium: grip strength <=20th percentile	categorical	calculated by hospital 2008
fragil	0,1,2	Frail status according to *Fried* scale	categorical	calculated by hospital 2008
ppeso_2013	0,1	Fried criterium: weight loss >10 lbs. in past yr	categorical	calculated by hospital 2013
exhaustion_2013	0,1	Fried criterium: exhaustion >=3days in past week	categorical	calculated by hospital 2013
pasefrag_2013	0,1	Fried criterium: PASE <=20 percentile	categorical	calculated by hospital 2013
marchafragil_2013	0,1	Fried criterium: time to walk >=80th percentile	categorical	calculated by hospital 2013
fuerzafragil_2013	0,1	Fried criterium: grip strength <=20th percentile	categorical	calculated by hospital 2013
fragil2013	0,1,2	Frail status according to *Fried* scale	categorical	calculated by hospital 2013

Table A.1: Features from the data set related to the *Fried* questions for determining the frailty status.

Attribute name	Values expected	Description	Type	How and when was it recorded?
ys1	yes, no	GDS1:Are you basically satisfied with your life?	binary	questionnaire answered by patient 2008
ys2	yes, no	GDS2:Have you dropped many of your activities and interests?	binary	questionnaire answered by patient 2008
ys3	yes, no	GDS3:Do you feel that your life is empty?	binary	questionnaire answered by patient 2008
ys4	yes, no	GDS4:Do you often get bored?	binary	questionnaire answered by patient 2008
ys5	yes, no	GDS5:Are you in good spirits most of the time?	binary	questionnaire answered by patient 2008
ys6	yes, no	GDS6:Are you afraid that something bad is going to happen to you?	binary	questionnaire answered by patient 2008
ys7	yes, no	GDS7:Do you feel happy most of the time?	binary	questionnaire answered by patient 2008
ys8	yes, no	GDS8:Do you often feel helpless?	binary	questionnaire answered by patient 2008
ys9	yes, no	GDS9:Do you prefer to stay at home, rather than going out and doing new things?	binary	questionnaire answered by patient 2008
ys10	yes, no	GDS10:Do you feel you have more problems with memory than most?	binary	questionnaire answered by patient 2008
ys11	yes, no	GDS11:Do you think it is wonderful to be alive now?	binary	questionnaire answered by patient 2008
ys12	yes, no	GDS12:Do you feel pretty worthless the way you are now?	binary	questionnaire answered by patient 2008
ys13	yes, no	GDS13:Do you feel full of energy?	binary	questionnaire answered by patient 2008
ys14	yes, no	GDS14:Do you feel that your situation is hopeless?	binary	questionnaire answered by patient 2008
ys15	yes, no	GDS15:Do you think that most people are better off than you are?	binary	questionnaire answered by patient 2008
gdstotal	0-15	GDS: Total Score	numeric	calculated by hospital 2008
depression	yes,no	gdstotal>=5	binary	calculated by hospital 2008

Table A.2: Features from the data set related to the Geriatric Depression Scale (*GDS*) questionnaire.

Attribute name	Values expected	Description	Type	How and when was it recorded?
drug_*n*_comercial_name	Name	Drug *n* commercial name	text	hospital 2008
drug_*n*_pa	Drug name	Drug *n* Active drug	text	hospital 2008
drug_*n*_atc	Code	Drug *n* ATC code	text	hospital 2008
drug_*n*a	1,2,3,NAN	How do you take it? 1. continuous; 2. intermittent; 3. Sporadic; other NAN	categorical	hospital 2008
drug_*n*b	1,2,3,NAN	When did you start to take it? 1. less than 1 month; 2. from 1 month to 1 year; 3. more than 1 year; other. NAN	categorical	hospital 2008

Table A.3: Medication related features from the data set: there are 11 ($n = \{1...11\}$) different drug attribute sets. All have the same format as in this table.

**Attribute name**	**Values expected**	**Description**	**Type**	**How and when was it recorded?**	**Metabolic system**
p1leu	4.5-11	Leucocytes [x10^9/L]	numeric	laboratory 2008	immune
p2hema	4-5	Erythrocytes [x10^{12}/L]	numeric	laboratory 2008	erythrocytes
p3hgb	12-15	Hemoglobyn [g/dL]	numeric	laboratory 2008	erythrocytes
p4hct	37-47	Hematocrit [%]	numeric	laboratory 2008	erythrocytes
p5vcm	80-99	Mean Corpuscular Volume (MCV) [fL]	numeric	laboratory 2008	erythrocytes
p6hcm	27-31	Mean Corpuscular Haemoglobin (MCH) [pg]	numeric	laboratory 2008	erythrocytes
p7chcm	33-37	Mean Corpuscular Haemoglobin Concentration (CHCM) [g/dL]	numeric	laboratory 2008	erythrocytes
p8ade	11.5-14.5	Red Cell Distribution Width (RDW) [%]	numeric	laboratory 2008	erythrocytes
p9lin	1-5	Lymphocytes [x10^9/L]	numeric	laboratory 2008	immune
p10mono	0.4-1.3	Monocytes [x10^9/L]	numeric	laboratory 2008	immune
p13eos	0.02-0.6	Eosinophiles [x10^9/L]	numeric	laboratory 2008	immune
p14baso	0-0.2	Basophiles [x10^9/L]	numeric	laboratory 2008	immune
p15dd	<500	D Dimer [μg/L]	numeric	laboratory 2008	coagulation
p16plaq	120-400	Platelets [x10^9/L]	numeric	laboratory 2008	coagulation
p17vpm	7-12	Mean Platelet Volume (MPV) [fl]	numeric	laboratory 2008	coagulation
p23glu	60-100	Glucose [mg/dL]	numeric	laboratory 2008	sugars
p24urea	10-71	Urea [mg/dL]	numeric	laboratory 2008	nephritic
p25acur	2.4-5.7	Uric acid [mg/dL]	numeric	laboratory 2008	nephritic
p26crea	0.5-0.9	Creatinine [mg/dL]	numeric	laboratory 2008	nephritic
p27prot	6.4-8.3	Protein [g/dL]	numeric	laboratory 2008	proteins
p28albu	3.4-4.8	Albumin [g/dL]	numeric	laboratory 2008	proteins
p30chol	110-230	Cholesterin [mg/dL]	numeric	laboratory 2008	fats
p31trig	60-200	Triglycerides [mg/dL]	numeric	laboratory 2008	fats
p32ca	8.4-10.2	Calcium (Ca) [mg/dL]	numeric	laboratory 2008	minerals
p33p	2.7-4.5	Phosphorus (P) [mg/dL]	numeric	laboratory 2008	minerals
p34na	132-146	Sodium (Na) [mEq/L]	numeric	laboratory 2008	minerals
p35k	3.7-5.4	Potassium (K) [mEq/L]	numeric	laboratory 2008	minerals
p36cl	94-110	Chloride (Cl) [mEq/L]	numeric	laboratory 2008	minerals
p37got	5-37	Glutamic-Oxaloacetic Transaminase (GOT) [U/L]	numeric	laboratory 2008	hepatic
p38gpt	5-40	Glutamic-Pyruvic Transaminase (GPT) [U/L]	numeric	laboratory 2008	hepatic
p39ggt	5-39	Gamma-Glutamyl Transferase (GGT) [U/L]	numeric	laboratory 2008	hepatic
p40falc	35-104	Alkaline phosphatase [U/L]	numeric	laboratory 2008	hepatic / nephritic
p41ldh	230-530	Lactate dehydrogenase (LDH) [U/L]	numeric	laboratory 2008	general
p42fe	40-145	Iron (FE) [μg/dL]	numeric	laboratory 2008	minerals
p43tfrr	200-360	Transferrin [mg/dL]	numeric	laboratory 2008	general
p44pcrh	<9	High-sensitivity C-reactive protein (hs-CRP) [mg/L]	numeric	laboratory 2008	cardiac
IGF1	50-300	Insulin like growth factor 1 (IGF1) [ng/mL]	numeric	laboratory 2008	growth
E2	0-200	17β-estradiol (E2) [pmol/L]	numeric	laboratory 2008	hormones
Dheas	0-200	Dehydroepiandrosterone sulfate (DHEA-S) [μg/dL]	numeric	laboratory 2008	homones
Dhea	0-10	Dehydroepiandrosterone (DHEA) [ng/mL]	numeric	laboratory 2008	homones
HDL	0-200	High-density lipoprotein (HDL) [mg/dL]	numeric	laboratory 2008	fats
LDL	0-200	Low-density lipoprotein (LDL) [mg/dL]	numeric	laboratory 2008	fats
INSULINA	0-2000	Insulin [U/mL]	numeric	laboratory 2008	sugars
ADMA	50-150	Asymmetric dimethylarginine (ADMA) [μmol/L]	numeric	laboratory 2008	proteins
TESTOTOTAL	0-1000	Total testosterone [ng/dL]	numeric	laboratory 2008	homones
TESTOLIBRE	0-10	Free testosterone [ng/dL]	numeric	laboratory 2008	homones

Table A.4: Blood related features from the data set.

Attribute name	Values expected	Description	Type	How and when was it recorded?
k1	111,222,333	WHO activity 6: Any difficulty washing face and arms?	categorical	questionnaire answered by patient 2008
k2	111,222,333	WHO activity 8: Any difficulty dressing and undressing?	categorical	questionnaire answered by patient 2008
k3	111,222,333	WHO activity 11: Any difficulty using the toilet?	categorical	questionnaire answered by patient 2008
k4	111,222,333	WHO activity 12: Any difficulty getting in and out of bed?	categorical	questionnaire answered by patient 2008
k5	111,222,333	WHO activity 19: Any difficulty controlling urination and bowel movements?	categorical	questionnaire answered by patient 2008
k6	111,222,333	WHO activity 9: Any difficulty eating (e.g.,holding a fork, cutting food, drinking from a glass)?	categorical	questionnaire answered by patient 2008
katz2008	0-6	Number of ADL abilities	numeric	calculated by hospital 2008
k1_2013	1,2,3	WHO activity 6: Any difficulty washing face and arms?	categorical	questionnaire answered by patient 2013
k2_2013	1,2,3	WHO activity 8: Any difficulty dressing and undressing?	categorical	questionnaire answered by patient 2013
k3_2013	1,2,3	WHO activity 11: Any difficulty using the toilet?	categorical	questionnaire answered by patient 2013
k4_2013	1,2,3	WHO activity 12: Any difficulty getting in and out of bed?	categorical	questionnaire answered by patient 2013
k5_2013	1,2,3	WHO activity 19: Any difficulty controlling urination and bowel movements?	categorical	questionnaire answered by patient 2013
k6_2013	1,2,3	WHO activity 9: Any difficulty eating (e.g.,holding a fork, cutting food, drinking from a glass)?	categorical	questionnaire answered by patient 2013
katz2013	0-6	Number of ADL abilities	categorical	calculated by hospital 2013

Table A.5: Activities of Daily Living questionnaire (*ADL*) related features from the data set.

Attribute name	Values expected	Description	Type	How and when was it recorded?
lw1	111,222,333,444	WHO activity 20: Any difficulty using the telephone?	categorical	questionnaire answered by patient 2008
lw2	111,222,333,444	WHO activity 5: Any difficulty shopping daily for basic necessities?	categorical	questionnaire answered by patient 2008
lw3	111,222,333,444	WHO activity 10: Any difficulty cooking a simple meal?	categorical	questionnaire answered by patient 2008
lw4	111,222,333,444,555	WHO activity 13: Any difficulty doing light housework (e.g., doing dishes, light cleaning)?	categorical	questionnaire answered by patient 2008
lw5	111,222,333	WHO activity 14: Any difficulty doing heavy housework (e.g., washing windows, floor)?	categorical	questionnaire answered by patient 2008
lw6	111,222,333,444,555	WHO activity 22: Any difficulty using public transportation?	categorical	questionnaire answered by patient 2008
lw7	111,222,333	WHO activity 23: Any difficulty taking medications correctly?	categorical	questionnaire answered by patient 2008
lw8	111,222,333	WHO activity 24: Any difficulty managing home finances?	categorical	questionnaire answered by patient 2008
lawton2008	0-8	Number of IADL abilities (0-8)	numeric	calculated by hospital 2008
lw1_2013	1,2,3,4	WHO activity 20: Any difficulty using the telephone?	categorical	questionnaire answered by patient 2013
lw2_2013	1,2,3,4	WHO activity 5: Any difficulty shopping daily for basic necessities?	categorical	questionnaire answered by patient 2013
lw3_2013	1,2,3,4	WHO activity 10: Any difficulty cooking a simple meal?	categorical	questionnaire answered by patient 2013
lw4_2013	1,2,3,4,5	WHO activity 13: Any difficulty doing light housework (e.g., doing dishes, light cleaning)?	categorical	questionnaire answered by patient 2013
lw5_2013	1,2,3	WHO activity 14: Any difficulty doing heavy housework (e.g., washing windows, floor)?	categorical	questionnaire answered by patient 2013
lw6_2013	1,2,3,4,5	WHO activity 22: Any difficulty using public transportation?	categorical	questionnaire answered by patient 2013
lw7_2013	1,2,3	WHO activity 23: Any difficulty taking medications correctly?	categorical	questionnaire answered by patient 2013
lw8_2013	1,2,3	WHO activity 24: Any difficulty managing home finances?	categorical	questionnaire answered by patient 2013
lawton2013	0-8	Number of IADL abilities	categorical	physician 2013

Table A.6: Instrumental Activities of Daily Living ($IADL$) questionnaire related features from the data set

Attribute name	Values expected	Description	Type	How and when was it recorded?
alch1	0-8	How many drinks do you have?	categorical	questionnaire answered by patient 2008
alch1a1	ℕ+	how many glasses of wine do you drink daily?	numeric	questionnaire answered by patient 2008
alch1a2	ℕ+	how many glasses of beer do you drink daily?	numeric	questionnaire answered by patient 2008
alch1a3	ℕ+	how many glasses of spirits do you drink daily?	numeric	questionnaire answered by patient 2008
alch1b	0-age	For how many years?	numeric	questionnaire answered by patient 2008
alch2	yes,no	did you drink previously?	binary	questionnaire answered by patient 2008
alch2a	1-5	Kind of drinker	categorical	questionnaire answered by patient 2008
alch2b	1-8	Starting age	categorical	questionnaire answered by patient 2008
alch2c	1-8	Ending age	categorical	questionnaire answered by patient 2008
tab1	1-4	Have you smoked at least 100 cigarettes in your entire life?	categorical	questionnaire answered by patient 2008
tab1a	1-3	If yes, Did you smoke cigarettes daily, occasionally, or not at all?	categorical	questionnaire answered by patient 2008
tab1a1	1-3	Do you smoke actually?	categorical	questionnaire answered by patient 2008
tab1a1a	1-8	If not, How many time have you stopped smoking?	categorical	questionnaire answered by patient 2008
tab1a3	0-age	For how many years did you smoke?	numeric	questionnaire answered by patient 2008
@1_year_smoker	yes, no	smoker for at least one year	binary	physician 2008
current_smoker	yes, no	current smoker	binary	physician 2008

Table A.7: Consumption related features from the data set.

Attribute name	Values expected	Description	Type	How and when was it recorded?
fuerza1a	0-60	Muscle strength (upper) with dynamometer: hand grip dominant limb (kg)	numeric	physician 2008
peso1	30-200	Weight (kg)	numeric	physician 2008
altura1	120-220	Height (cm)	numeric	physician 2008
ppca	20-200	Anthropometry: hip perimeter (cm)	numeric	physician 2008
pasetotal	0-400+	Physical activity scale for elderly score	numeric	questionnaire answered by patient 2008
codigo01	alive,death	Dead at follow up?	binary	physician 2008

Table A.8: Physique related features from the data set.

Attribute name	Values expected	Description	Type	How and when was it recorded?
ccv1	yes,no	Myocardial infarction / Heart attack (self reported)+E148	binary	questionnaire answered by patient 2008
ccv2	yes,no	Congestive heart failure (self reported)	binary	questionnaire answered by patient 2008
ccv4	yes,no	Angina pectoris (self reported)	binary	questionnaire answered by patient 2008
ccv6	yes,no	Hypertension (self-report,drugs,BP tests)	binary	questionnaire answered by patient 2008
ccv8	yes,no	Diabetes mellitus (self reported, drugs)	binary	questionnaire answered by patient 2008
cv1cv4	yes,no	Myocardial infarction / Heart attack (self reported)/angina pectoris	binary	questionnaire answered by patient 2008
charlsonindex	0-37	Charlson co-morbidity index	categorical	physician 2008

Table A.9: Comorbidity related features from the data set.

Attribute name	Values expected	Description	Type	How and when was it recorded?
enpot1	0,1,NaN	What day of the week is this? (MMSE question)	categorical	questionnaire answered by patient 2008
enpot2	0,1,NaN	What is today's date? (MMSE question)	categorical	questionnaire answered by patient 2008
enpot3	0,1,NaN	What month is this? (MMSE question)	categorical	questionnaire answered by patient 2008
enpot4	0,1,NaN	What year is this? (MMSE question)	categorical	questionnaire answered by patient 2008
enpot6	0,1,NaN	Which season is this? (MMSE question)	categorical	questionnaire answered by patient 2008
enpol1	0,1,NaN	IN HOME: What is the street address of this house? // IN FACILITY: What is the name of this building? (MMSE question)	categorical	questionnaire answered by patient 2008
enpol2	0,1,NaN	IN HOME: What room are we in? // IN FACILITY: What floor are we on? (MMSE question)	categorical	questionnaire answered by patient 2008
enpol3	0,1,NaN	What city/town are we in? (MMSE question)	categorical	questionnaire answered by patient 2008
enpol4	0,1,NaN	What province are we in? (MMSE question)	categorical	questionnaire answered by patient 2008
enpol5	0,1,NaN	What county are we in? (MMSE question)	categorical	questionnaire answered by patient 2008
enpmem1a	1,2,3,4,NaN	SAY: I am going to name three objects. When I am finished, I want you to repeat theM. Remember what they are because I am going to ask you to name them again in a few minutes. // Say the following words slowly at 1-second intervals - peseta (coin in spanish), caballo (horse in spanish), manzana (apple in spanish) (MMSE question)	categorical	questionnaire answered by patient 2008
enpat2	1,2,3,4,5,6,NaN	Spell the word MUNDO (world in spanish). Now spell it backwards.	categorical	questionnaire answered by patient 2008
enpat1	1,2,3,4,5,6,NaN	Count backwards by 7 starting from 100	categorical	questionnaire answered by patient 2008
enpmem2	1,2,3,4,NaN	Now what were the three objects I asked you to remember?	categorical	questionnaire answered by patient 2008
enpleng1	1,2,3,NaN	Show a wristchatch and a pencil. What are these called?	categorical	questionnaire answered by patient 2008
enpleng2	1,2,NaN	SAY: I would like you to repeat this phrase after me: Ni si, ni no, ni pero. (No ifs, ands or buts. In spanish)	categorical	questionnaire answered by patient 2008
enpleng4	1,2,NaN	SAY: Read the words on the page and then do what it says. Then hand the person the sheet with "Cierre los ojos" (close your eyes in spanish) on it. If the subject read and does not close their eyes, repeat yp to three times. Score only if subject closes eyes.	categorical	questionnaire answered by patient 2008
enpprx1	1,2,NaN	Hand the person a pencil and paper. SAY: write any complete sentence on that piece of paper. (Note: The sentence must make sense. Ignore spelling errors)	categorical	questionnaire answered by patient 2008
enpprx2	1,2,NaN	Place design, eraser and pencil in front of the person. SAY: copy this design please. // Allow multiple tries. Wait until person is finished and hands it back. Score only for correctly copied diagram with a 4-sided figure between two 5-sided figures.	categorical	questionnaire answered by patient 2008
enpleng3	1,2,3,4,NaN	Ask the person if he is right or left handed. Take a piece of paper and hold it up in front of the person. SAY: Take this paper in your right/left hand (whichever is non-dominant), fold the paper in half once with both hands and put the paper down on the floor. Score 1 point for each instruction executed correctly.	categorical	questionnaire answered by patient 2008
mmse2009	0-30	MMSE raw score	numeric	calculated by hospital 2008
cognitive _impairment _mmse _educative _level	yes,no	Has the patient a cognitive impairment?	binary	determined by physician 2008

Table A.10: Mini-Mental-State-Examination ($MMSE$) related features from the data set.

Attribute name	Values expected	Description	Type	How and when was it recorded?
cq8	yes, no, 88 = NA	Leukemia or Polycytemia	categorical	physician 2008
cq9	yes, no, 88 = NA	Lymphoma	categorical	physician 2008
cq10	yes, no, 88 = NA	Cancer (except Leukemia, polycythemia and lymphoma)	categorical	physician 2008
cq6	1,2.3,4	Did any doctor tell you that you had Alzheimer's disease, senile dementia or another dementia?	categorical	questionnaire answered by patient 2008
cq6a	1-10	What kind of dementia did your say doctor that you had?	categorical	questionnaire answered by patient 2008
reum1	1,2.3,4	Have you ever had any joint inflammated for more than 4 weeks in a row?	categorical	questionnaire answered by patient 2008
reum2	1,2.3,4	Have you ever felt pain in any joint for more than 4 weeks in a row?	categorical	questionnaire answered by patient 2008
reum3	1,2.3,4	Do you ever feel that you can't move or feel rigid for over half an hour during the morning?	categorical	questionnaire answered by patient 2008
reum4	1,2.3,4	Have you ever been told you have arthritis?	categorical	questionnaire answered by patient 2008
reum5	1-9	Please select in the mannequin the joints in which you have had or have now inflammation for more than 4 weeks in a row (note the location of the affected joints). SHOW CARD 2.	categorical	questionnaire answered by patient 2008
reum6	1,2.3,4	Do you feel pain or have inflammation in any joint?	categorical	questionnaire answered by patient 2008
reum6a	1-9	If yes,Please, show which joints. SHOW CARD 2:	categorical	questionnaire answered by patient 2008
reum7	1-6	Did any doctor tell you that you had arthritis or arthrosis in your..?	categorical	questionnaire answered by patient 2008
reum7a	1,2.3,4	if yes (1, 2 or 3)The doctor said that you had it after a hip or knee radiography, or both?	categorical	questionnaire answered by patient 2008
epoc1	1,2.3,4	Did any doctor tell you that you had a chronic obstructive pulmonary disease: emphysema or chronic bronchitis?	categorical	questionnaire answered by patient 2008
epoc2	1,2.3,4	Did any doctor say tell that you had asthma?	categorical	questionnaire answered by patient 2008
epoc3	1,2.3,4	Did any doctor tell you that you had any lung disease?	categorical	questionnaire answered by patient 2008
epoc4	1,2.3,4	Did any doctor tell you that you had had a pneumonía or bronchopneumonía?	categorical	questionnaire answered by patient 2008
epoc5	1,2.3,4	Did any doctor tell you that you had had an acute bronchitis?	categorical	questionnaire answered by patient 2008
epoc6	1,2.3,4	Have you ever been operated of your lung?	categorical	questionnaire answered by patient 2008
epoc7	1,2.3,4	Do you have any other lung disease?	categorical	questionnaire answered by patient 2008

Table A.11: Disease related features from the data set

Attribute name	Values expected	Description	Type	How and when was it recorded?
em1	yes,no	Are you able to walk at home?	binary	questionnaire answered by patient 2008
em1a	yes,no	If answered YES; Do you get tired when doing it?	binary	questionnaire answered by patient 2008
em1b	yes,no	If answered YES; Do you need help when doing it?	binary	questionnaire answered by patient 2008
em2	yes,no	Are you able to go out from home?	binary	questionnaire answered by patient 2008
em2a	yes,no	If answered YES; Do you get tired when doing it?	binary	questionnaire answered by patient 2008
em2b	yes,no	If answered YES; Do you need help when doing it?	binary	questionnaire answered by patient 2008
em3	yes,no	Are you able to climb stairs?	binary	questionnaire answered by patient 2008
em3a	yes,no	If answered YES; Do you get tired when doing it?	binary	questionnaire answered by patient 2008
em3b	yes,no	If answered YES; Do you need help when doing it?	binary	questionnaire answered by patient 2008
em4	yes,no	Are you able to walk outside (nice weather)?	binary	questionnaire answered by patient 2008
em4a	yes,no	If answered YES; Do you get tired when doing it?	binary	questionnaire answered by patient 2008
em4b	yes,no	If answered YES; Do you need help when doing it?	binary	questionnaire answered by patient 2008
em5	yes,no	Are you able to walk outside (bad weather)?	binary	questionnaire answered by patient 2008
em5a	yes,no	If answered YES; Do you get tired when doing it?	binary	questionnaire answered by patient 2008
em5b	yes,no	If answered YES; Do you need help when doing it?	binary	questionnaire answered by patient 2008

Table A.12: Mobility Scale (MS) related features from the data set.

Attribute name	Values expected	Description	Type	How and when was it recorded?
hi1	7-8 digit number	ETES ID	numeric	assigned and recorded by the hospital
frailomic_code	"TO" + hi1	FRAILOMIC ID	2 constant characters + numeric	assigned and recorded by the hospital
Parma_serum_code	Code	Parma Serum code	text	assigned and recorded by the hospital
Parma_Edta_code	Code	Parma EDTA Code	text	assigned and recorded by the hospital
Jena_Edta_code	Code	Jena EDTA Code	text	assigned and recorded by the hospital
Evercyte_Edta_code	Code	Evercyte EDTA Code	text	assigned and recorded by the hospital
Cardiff_serum_code	Code	Cardiff Serum Code	text	assigned and recorded by the hospital
Cardiff_Edta_code	Code	Cardiff EDTA Code	text	assigned and recorded by the hospital
EV_Edta_code	Code	EV EDTA Code	text	assigned and recorded by the hospital

Table A.13: Codes and IDs of the hospital which appear in the data set.

Attribute name	Values expected	Description	Type	How and when was it recorded?
hi8	0-130	Age in years	numeric	physician 2008
hi11	male, female	Gender	binary	physician 2008
individualincome	1-12	Income of the individual	categorical	physician 2008
householdincome	1-15	Income of the household in which the individual lives	categorical	physician 2008
numpersonsfamilyunit	1-10	Number of persons in the family	categorical	physician 2008

Table A.14: Features related to demographic properties of the patients.

Attribute name	Values expected	Description	Type	How and when was it recorded?
ekg1	40-200	EKG: Heart rate (beats/minute)	numeric	physician 2008
tadd	40-140	Pressure arterial. Diastolic	numeric	physician 2008
tads	80-260	Pressure arterial. Systolic	numeric	physician 2008

Table A.15: Features related to cardiac properties of the patients.

Attribute name	Values expected	Description	Type	How and when was it recorded?
ps1	1-6	How would you evaluate your current health? How do you feel now?	categorical	questionnaire answered by patient 2008
ps2	1-6	How is your health compared to 1 yr ago?	categorical	questionnaire answered by patient 2008
ps3	1-6	How would you judge your health compared to other people of your same age?	categorical	questionnaire answered by patient 2008
ps4	1-6	Are you happy in general?	categorical	questionnaire answered by patient 2008
ps5	1-6	If you are thinking about you life till now, how satisfied are you?	categorical	questionnaire answered by patient 2008
ps6	1-6	Are you feeling incapable of tackling problems in your life?	categorical	questionnaire answered by patient 2008
ps7	1-6	Are you feel capable of tackling every task you would like to?	categorical	questionnaire answered by patient 2008
ps8	1-5	During the last week, did you feel physical pain?	categorical	questionnaire answered by patient 2008
ps9	1-5	During the last month, how many times did you visit the general practitioner because of being sick?	categorical	questionnaire answered by patient 2008
ps10	1-6	When was the last time that you visited a medical doctor or another medical professional in order to speak about your health?	categorical	questionnaire answered by patient 2008
ps11	1-7	Think about the most painful/woebegone event which has happened in the last ten years. How much time did you need to recover from it?	categorical	questionnaire answered by patient 2008
ps12	1-5	During the last 12 months, how many times have you been hospitalized (over night)?	categorical	questionnaire answered by patient 2008
ps13	1-7	During the last 12 months, how many times did you visit the hospital because of an emergency (without spending the night)?	categorical	questionnaire answered by patient 2008
ps14	0,1	During the last 12 months, where you patient in a rehabilitation center (with spending the night)?	binary	questionnaire answered by patient 2008
ps14a	1-6	During the last 12 months, how much time did you spend in an institution for physical therapy (with spending the night)?	categorical	questionnaire answered by patient 2008

Table A.16: Features related to self reported health status of the patients.
